# Clinical impact of stress dose steroids in patients with septic shock: insights from the PROWESS-Shock trial

**DOI:** 10.1186/s13054-015-0921-x

**Published:** 2015-04-28

**Authors:** Pedro Póvoa, Jorge I F Salluh, Maria L Martinez, Raquel Guillamat-Prats, Dianne Gallup, Hussein R Al-Khalidi, B Taylor Thompson, V Marco Ranieri, Antonio Artigas

**Affiliations:** Polyvalent Intensive Care Unit, São Francisco Xavier Hospital, Centro Hospitalar de Lisboa Ocidental, Lisbon, Portugal; NOVA Medical School, CEDOC, New University of Lisbon, Lisbon, Portugal; D’or Institute for Research and Education, Rio de Janeiro, Brazil; Postgraduation Program, Instituto Nacional de Câncer, Rio de Janeiro, Brazil; Critical Care Center, Sabadell Hospital, Corporación Sanitaria Universitaria Parc Taulí, Universitat Autonoma de Barcelona, Sabadell, Spain; CIBER de Enfermedades Respiratorias (CIBERES), Madrid, Spain; Duke Clinical Research Institute, Durham, NC USA; Pulmonary and Critical Care Unit, Department of Medicine, Massachusetts General Hospital, Boston, USA; Dipartimento di Anestesiologia e Rianimazione, Azienda Ospedaliera Città della Salute e della Scienza e di Torino_Molinette, Università di Torino, Torino, Italy

## Abstract

**Introduction:**

The aim of our study was to evaluate the clinical impact of the administration of intravenous steroids, alone or in conjunction with drotrecogin-alfa (activated) (DrotAA), on the outcomes in septic shock patients.

**Methods:**

We performed a sub-study of the PROWESS-Shock trial (septic shock patients who received fluids and vasopressors above a predefined threshold for at least 4 hours were randomized to receive either DrotAA or placebo for 96 hours). A propensity score for the administration of intravenous steroids for septic shock at baseline was constructed using multivariable logistic regression. Cox proportional hazards model using inverse probability of treatment weighting of the propensity score was used to estimate the effect of intravenous steroids, alone or in conjunction with DrotAA, on 28-day and 90-day all-cause mortality.

**Results:**

A total of 1695 patients were enrolled of which 49.5% received intravenous steroids for treatment of septic shock at baseline (DrotAA + steroids N = 436; DrotAA + no steroids N = 414; placebo + steroids N = 403; placebo + no steroids N = 442). The propensity weighted risk of 28-day as well as 90-day mortality in those treated vs. those not treated with steroids did not differ among those randomized to DrotAA vs. placebo (interaction p-value = 0.38 and p = 0.27, respectively) nor was a difference detected within each randomized treatment. Similarly, the course of vasopressor use and cardiovascular SOFA did not appear to be influenced by steroid therapy. In patients with lung infection (N = 744), abdominal infection (N = 510), Gram-positive sepsis (N = 420) and Gram-negative sepsis (N = 461), the propensity weighted risk of 28-day as well as 90-day mortality in those treated vs. those not treated with steroids did not differ among those randomized to DrotAA vs. placebo nor was a difference detected within each randomized treatment.

**Conclusions:**

In the present study of septic shock patients, after adjustment for treatment selection bias, we were unable to find noticeable positive impact from intravenous steroids for treatment of septic shock at baseline either in patients randomized for DrotAA or placebo.

**Trial registration:**

Clinicaltrials.gov NCT00604214. Registered 24 January 2008.

## Introduction

Severe sepsis and septic shock are amongst the major causes of intensive care admissions [1,2] and despite the recent improvements in clinical outcomes, mortality rates are still elevated, varying from 20 to 35% [3-5]. Improved outcome is mainly ascribed to earlier identification and improvements in the process of care of sepsis rather than specific pharmacologic interventions [6-9].

In recent years, the uses of corticosteroids and to a lesser degree drotrecogin-alfa activated (DrotAA) have been the cornerstones of adjunctive pharmacologic therapy for severe sepsis and septic shock [10-13]. However, the results of the more recent clinical trials have failed to demonstrate clinical benefits from either intervention [14-16]. In addition, supporters of the use of corticosteroids for septic shock claim that the CORTICUS study results had limited external validity due to the fact that it excluded patients whose clinicians decided to treat with corticosteroids. This a priori decision potentially biased the study by enroling patients with either lower severity of illness or those thought to receive less benefit [17,18]. Furthermore, although there are potential synergies in the concomitant use of corticosteroids and DrotAA, only one recent study evaluated this issue, and it was limited by the discontinuation of the DrotAA arm when the drug was withdrawn from the market [16].

In the present study, we have evaluated the clinical impact of corticosteroids alone or in conjunction with DrotAA in patients with septic shock by analyzing data from the PROWESS-Shock trial [15]. We hypothesized that the patients who received corticosteroids as part of usual care will improve their outcomes after adjustment for baseline imbalances.

## Materials and methods

### Study design and setting

Patients diagnosed with septic shock were randomly assigned to receive either DrotAA (24 μg/kg/hour) or placebo administered intravenously for 96 hours [15]. All details of the PROWESS-Shock trial and its design have been reported elsewhere (NCT00604214) [15]. The PROWESS-Shock trial was approved by the research ethics boards of all participating institutions. Patients, next of kin, or surrogate decision-makers gave written informed consent in accordance with local requirements. The trial was conducted in accordance with the Declaration of Helsinki. The present analysis was proposed and approved by the Steering Committee of the PROWESS-Shock trial, which in addition considered there was no need for further ethical approval.

The concomitant use of steroids as adjunctive treatment of septic shock, according to the recommendations at the time [13], was at the discretion of the attending physician and was not required by the study protocol. The question used in the case report form to collect data on steroid use was the following: “Was the subject treated with any intravenous steroid therapy for septic shock during the pretreatment period (before study drug infusion)?” No additional information on steroid adjunctive therapy use was recorded, namely type of steroid, dose, type of infusion (intermittent or continuous) or duration of therapy.

For the present analysis we divided the two arms of the trial, the DrotAA and the placebo arms, into four groups according to the prescription of intravenous steroid therapy for septic shock. These were: 1) patients receiving steroids at baseline and randomized to receive DrotAA; 2) patients not receiving steroids at baseline and randomized to receive DrotAA; 3) patients receiving steroids at baseline and randomized to receive placebo, and 4) patients not receiving steroids at baseline and randomized to receive placebo. Study outcome was all-cause mortality at 28 days and at 90 days. In addition, we assessed the course of organ failure, in particular cardiovascular sequential organ failure assessment (SOFA) score, as well as the mortality due to secondary refractory septic shock.

### Statistical analysis

Baseline characteristics of patients were compared by treatment strategy using the Kruskal-Wallis test for continuous variables and Pearson Chi-square test for categorical variables. Kaplan-Meier estimates and survival curves for 28-day and 90-day all-cause mortality amongst all patients were weighted by the inverse probability of receiving steroid therapy at baseline, using a propensity score.

Estimates of the hazard ratios (95% CI) of all-cause mortality for all patients and for patients within each of the following subgroups were displayed in a forest plot on the log-odds scale in: 1) patients with lung infection; 2) patients with abdominal infection; 3) patients with Gram-positive sepsis, and 4) patients with Gram-negative sepsis. The hazard ratio (95% CIs) estimates and the *P*-values for the interaction terms of randomized treatment*baseline steroid use were obtained using inverse probability-weighted Cox proportional hazards models [19].

A propensity score (that is, probability of receiving intravenous steroid therapy for septic shock at baseline) was calculated using a multivariable logistic regression model after adjusting for clinically relevant patient characteristics at baseline. The following variables were selected to be included in the propensity model: age; gender; baseline acute physiology and chronic health evaluation (APACHE) II score; baseline total SOFA score; time between first vasopressor and study drug infusion (hours); number of baseline organ dysfunctions (1 to 5); baseline lactate concentration (mmol/L); IV fluids in 24 hours before start of vasopressors (mL); intravenous (IV) fluids from the start of vasopressor to the start of study drug infusion (mL); baseline vasopressor score (a dimensionless variable calculated as follows: dopamine dose (mcg/kg/min) × 1) + (dobutamine dose (mcg/kg/min) × 1) + (epinephrine dose (mcg/kg/min) × 100) + (norepinephrine dose (mcg/kg/min) × 100) + (phenylephrine dose (mcg/kg/min) × 100 + (vasopressin dose (mcg/kg/min) × 100) [20-22]; whether or not the patient had an abdominal infection, and whether or not the patient had a lung infection. The adequacy of the propensity model was assessed by checking the distribution of the propensity scores by treatment for a reasonable overlap and the pre- and post-inverse probability of treatment-weighting balance of the covariates [23].

Missing data were handled using the Markov chain Monte Carlo full-imputation strategy with a single imputation. After imputation, continuous variables were evaluated for linearity and cubic splines utilized, if necessary. Descriptive statistics for change in vasopressor-free days from day 1 to day 6 and change in cardiovascular SOFA from baseline to day 6 were provided. No statistical testing was performed to detect differences in these outcomes across treatment strategy because no information was obtained during the trial on when steroid treatment began post-baseline. *P*-values <0.05 were used to determine statistical significance. All analyses were performed using SAS version 9.2 (SAS Institute Inc., Cary, NC, USA).

## Results

In the PROWESS-Shock trial, 49.5% of patients received intravenous steroids for treatment of septic shock at baseline in the pretreatment period before DrotAA infusion (steroid use at baseline + DrotAA, n = 436; no steroid use at baseline + DrotAA, n = 414; steroid use at baseline + placebo, n = 403; no steroid use at baseline + placebo, n = 442; total n = 1,695. There was a difference of two patients from the enroled patients in the PROWESS-Shock trial, one patient was randomized without prior consent and another without baseline steroid usage information) [15].

Table 1 presents the baseline characteristics of the patients at trial inclusion according to the four defined groups. Patients who received intravenous steroids had a significantly higher APACHE II score, total SOFA score, need for mechanical ventilation and incidence of acute respiratory distress syndrome, and higher need for renal replacement therapy than patients who did not receive intravenous steroids in both arms of the trial. In addition, patients on steroids had significantly greater need of vasopressors, as indicated by a higher vasopressor score. Patients treated with steroids received a significantly lower volume of fluids in the 24 hours before start of vasopressors, but from the start of vasopressors to the start of the study drug (either DrotAA or placebo) they received a significantly greater volume. Finally, the median total amount of fluid received by each of the four groups was comparable (DrotAA with and without steroids, 8,130 and 8,238 mL, respectively; placebo with and without steroids, 8,000 and 8,065 mL, respectively, *P* = 0.69).

**Table 1:** **Baseline characteristics by steroid use for treatment of septic shock at baseline and randomized treatment of all patients**

	**DrotAA and steroid use**	**DrotAA and no steroid use**	**Placebo and steroid use**	**Placebo and no steroid use**	***P*** **-value** ^**a**^
Number	436	414	403	442	
Age, years					0.1007
Median (25th, 75th percentile)	64.4 (52.5, 74.2)	66.2 (55.4, 76.0)	66.2 (54.5, 76.6)	63.6 (51.4, 75.2)	
Female, n/total (%)	190/436 (43.6%)	169/414 (40.8%)	185/403 (45.9%)	194/442 (43.9%)	0.5350
Prior or preexisting conditions					
Alcohol dependence	58/436 (13.3%)	59/414 (14.3%)	53/403 (13.2%)	61/442 (13.8%)	0.9604
Chronic liver disease	18/436 (4.1%)	11/414 (2.7%)	12/403 (3.0%)	17/442 (3.8%)	0.6109
Chronic obstructive airways disease	69/436 (15.8%)	59/414 (14.3%)	66/403 (16.4%)	65/442 (14.7%)	0.8084
Chronic renal disease	49/436 (11.2%)	35/414 (8.5%)	30/403 (7.4%)	37/442 (8.4%)	0.2380
Congestive heart failure	27/436 (6.2%)	21/414 (5.1%)	22/403 (5.5%)	23/442 (5.2%)	0.8837
Coronary artery disease	57/436 (13.1%)	55/414 (13.3%)	37/403 (9.2%)	49/442 (11.1%)	0.2024
Diabetes mellitus	100/436 (22.9%)	89/414 (21.5%)	90/403 (22.3%)	126/442 (28.5%)	0.0618
Hypertension	190/436 (43.6%)	191/414 (46.1%)	201/403 (49.9%)	201/442 (45.5%)	0.3341
Immunodeficiency	40/436 (9.2%)	11/414 (2.7%)	41/403 (10.2%)	18/442 (4.1%)	<0.0001
Malignancy (cancer)	81/436 (18.6%)	64/414 (15.5%)	83/403 (20.6%)	64/442 (14.5%)	0.0629
Pancreatitis	18/436 (4.1%)	12/414 (2.9%)	13/403 (3.2%)	12/442 (2.7%)	0.6509
Stroke	25/436 (5.7%)	23/414 (5.6%)	19/403 (4.7%)	25/442 (5.7%)	0.9048
Positive blood culture	143/436 (32.8%)	127/414 (30.7%)	122/403 (30.3%)	117/442 (26.5%)	0.2262
Source control of infection^b^	138/151 (91.4%)	137/152 (90.1%)	133/149 (89.3%)	131/146 (89.7%)	0.9355
Number of baseline organ dysfunctions					<0.0001
1	8/436 (1.8%)	10/414 (2.4%)	6/403 (1.5%)	17/442 (3.8%)	
2	41/436 (9.4%)	74/414 (17.9%)	35/403 (8.7%)	77/442 (17.4%)	
3	133/436 (30.5%)	144/414 (34.8%)	132/403 (32.8%)	162/442 (36.7%)	
4	185/436 (42.4%)	142/414 (34.3%)	162/403 (40.2%)	154/442 (34.8%)	
5	69/436 (15.8%)	44/414 (10.6%)	68/403 (16.9%)	32/442 (7.2%)	
Time from start of vasopressor to infusion start, hours					0.1390
Median (25th, 75th percentile)	19.1 (13.0, 22.7)	17.0 (13.0, 21.5)	18.2 (12.8, 22.0)	18.0 (11.6, 22.2)	
Apache II score					<0.0001
Median (25th, 75th percentile)	25.0 (20.0, 31.0)	24.0 (19.0, 30.0)	26.0 (21.0, 32.0)	23.0 (18.0, 29.0)	
Recent surgery	159/436 (36.5%)	157/414 (37.9%)	143/403 (35.5%)	165/442 (37.3%)	0.8968
Mechanical ventilation	379/436 (86.9%)	316/414 (76.3%)	361/403 (89.6%)	339/442 (76.7%)	<0.0001
Renal replacement therapy	88/432 (20.4%)	32/413 (7.7%)	80/402 (19.9%)	27/442 (6.1%)	<0.0001
Acute respiratory distress syndrome	144/436 (33.0%)	80/414 (19.3%)	123/403 (30.5%)	114/442 (25.8%)	<0.0001
Intravenous fluids from start of vasopressor to start of study drug, mL					0.0093
Median (25th, 75th percentile)	4858 (3210, 7840)	4458 (2746, 6865)	4635 (2904, 7508)	4222 (2755, 6460)	
Intravenous fluids in 24 hours before start of vasopressors, mL					<0.0001
Median (25th, 75th percentile)	2902 (1850, 4384)	3250 (2197, 5100)	2850 (1600, 4350)	3250 (2105, 5000)	
Vasopressor score					<0.0001
Median (25th, 75th percentile)	50.0 (23.0, 98.0)	27.0 (12.2, 46.4)	50.0 (22.9, 90.9)	21.9 (12.6, 44.4)	
Total sequential organ failure assessment					<0.0001
Median (25th, 75th percentile)	11.0 (9.0, 13.0)	10.0 (8.0, 12.0)	11.0 (9.0, 13.0)	10.0 (8.0, 12.0)	
Lactate, mmol/L					<0.0001
Median (25th, 75th percentile)	3.0 (1.9, 4.8)	2.2 (1.4, 3.3)	3.1 (2.0, 5.1)	2.1 (1.4, 3.3)	

^a^Kruskal-Wallis *P*-value for continuous variables, chi-square test for categorical variables. ^b^Source control achieved/source control necessary. APACHE, acute physiology and chronic health evaluation; DrotAA, drotrecogin-alfa activated.

### Impact of steroids in septic shock mortality

**Figure 1:**
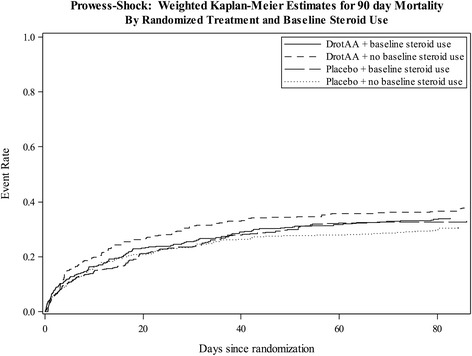
Propensity-weighted Kaplan-Meier 90-day all-cause mortality according to randomized treatment (DrotAA versus placebo) and baseline steroid use for septic shock treatment. *P* = 0.27. DrotAA, drotrecogin-alfa activated.

The propensity-weighted risk of 28-day and 90-day all-cause mortality in those treated with steroids versus those not treated with steroids at baseline did not differ among those randomized to DrotAA versus placebo (interaction *P*-value = 0.38 and *P* = 0.27, respectively) nor was a difference detected within each randomized treatment (Table 2). Figure 1 presents the weighted Kaplan-Meier 90-day mortality according to randomized treatment (DrotAA versus placebo) and baseline steroid use for septic shock treatment.

**Table 2:** **Survival analysis of 28-day and 90-day all-cause mortality (propensity-weighted), all patients, by steroid use at baseline and randomized treatment**

**Mortality**	**DrotAA and steroid use** ^**a**^	**DrotAA and no steroid use** ^**a**^	**Hazard ratio** ^**b**^	**Placebo and steroid use** ^**a**^	**Placebo and no steroid use** ^**a**^	**Hazard ratio** ^**b**^	**Interaction** ***P*** **-value** ^**b**^
**Day 28**							
Estimate	24.8%	29.5%	0.826	23.5%	23.3%	1.001	0.3764
95% CI	20.6, 29.7	25.1, 34.5	0.616, 1.107	19.2, 28.6	19.4, 27.8	0.735, 1.364	
**Day 90**							
Estimate	34.1%	37.9%	0.874	32.8%	30.6%	1.074	0.2743
95% CI	29.4, 39.3	33.1, 43.0	0.678, 1.127	27.9, 38.4	26.2, 35.4	0.822, 1.403	

^a^Inverse proportional weighted Kaplan-Meier rate and associated 95% CI. ^b^Hazard ratios of steroid versus no steroid use and randomized treatment*steroid use interaction *P*-value obtained using inverse proportional weighted Cox proportional hazards model. Observations weighted by the inverse probability of being prescribed a steroid therapy at baseline using a propensity model including the following variables: age, gender, baseline acute physiology and chronic health evaluation II score, baseline total sequential organ failure assessment, time between first vasopressor and study drug infusion (hours), number of baseline organ dysfunctions (1 to 5), baseline lactate concentration (mmol/L), intravenous (IV) fluids in 24 hours before start of vasopressors (mL), IV fluids from the start of vasopressor to the start of study drug infusion (mL), and baseline vasopressor score. DrotAA, drotrecogin-alfa activated.

**Figure 2:**
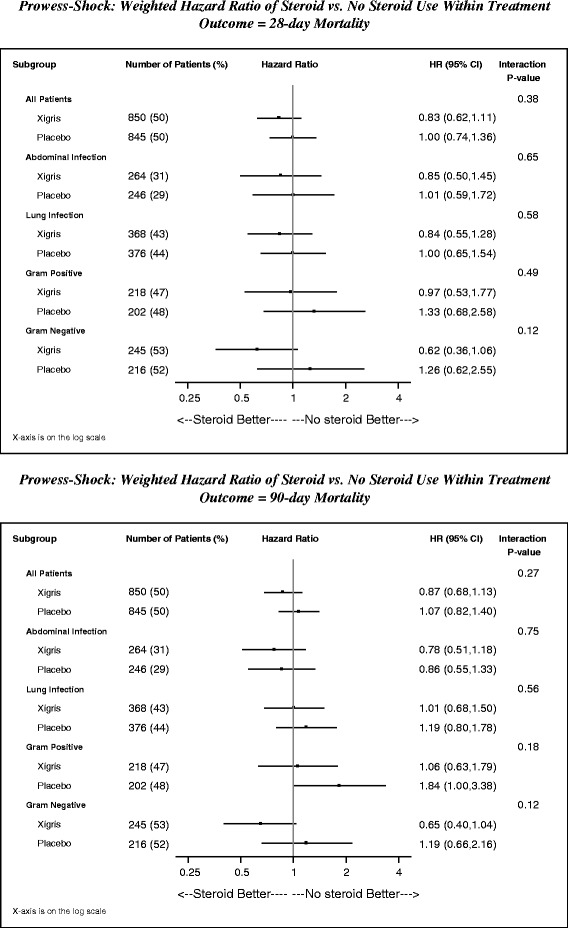
Impact of steroids in septic shock mortality; 28-day and 90-day all-cause mortality (propensity-weighted) for PROWESS-Shock patients and subgroups (abdominal infection, lung infection, Gram-positive infection and Gram-negative infection). D28, day 28; D90, day 90; HR, hazard ratio; DrotAA, drotrecogin-alfa activated.

In patients with lung infection (n = 744), patients with abdominal infection (n = 510), patients with Gram-positive sepsis (n = 420) and patients with Gram-negative sepsis (n = 461), the propensity-weighted hazard of 28-day as well as 90-day mortality in those treated with steroids versus those not treated with steroids did not differ for those randomized to DrotAA versus placebo nor was a difference detected within each randomized treatment (Figure 2).

### Impact of steroids in the course of septic shock

We described the effect of intravenous steroids in the weaning from vasopressor as expressed by propensity-weighted vasopressor-free days from day 1 to day 6 as well as propensity-weighted change of cardiovascular SOFA from baseline to day 6 (Table 3). Septic shock patients randomized to DrotAA or placebo who received steroids seemed to present a similar course of vasopressor use as those without steroid therapy. Likewise, there appeared to be a similar decrease in cardiovascular SOFA between those treated with steroids and those not treated with steroids, regardless of which randomized treatment was assigned, DrotAA or placebo. Finally, 90-day mortality (propensity-weighted) by refractory septic shock in patients randomized to DrotAA or placebo who received steroids was similar to those without steroid therapy (25.6, 28.8, 25.2 and 28.4%, respectively).

**Table 3:** **Course of septic shock (propensity-weighted) from baseline to day 6 by steroid use at baseline and randomized treatment**

	**DrotAA and steroid use**	**DrotAA and no steroid use**	**Placebo and steroid use**	**Placebo and no steroid use**
**Change in cardiovascular sequential organ failure assessment**				
Mean (SD)	−2.8 (1.6)	−2.7 (1.6)	−2.8 (1.6)	−2.9 (1.6)
Median (25th, 75th percentile)	−4 (−4,-1)	−4 (−4,-1)	−4 (−4,-1)	−4 (−4,-2)
Minimum, maximum	−4, 1	−4, 1	−4, 1	−4, 1
**Vasopressor-free days**				
Mean (SD)	2.5 (2.0)	2.7 (2.1)	2.4 (2.0)	2.9 (2.0)
Median (25th, 75th percentile)	3 (0,4)	3 (0,5)	3 (0,4)	4 (1,5)
Minimum, maximum	0, 6	0, 6	0, 6	0, 6

DrotAA, drotrecogin-alfa activated.

## Discussion

We found no benefits from the use of intravenous steroids for treatment of septic shock at baseline either in patients randomized to DrotAA or placebo. In addition, we observed that intravenous steroids did not seem to influence the clinical course of septic shock, expressed by the cardiovascular SOFA, vasopressor-free days, and death from refractory shock.

The role of steroids as an adjunctive therapy in the treatment of septic shock has been a controversial issue for many decades [24]. A large meta-analysis including 17 randomized controlled trials (RCT) and 3 quasi-RCTs suggested some survival benefit of prolonged low-dose corticosteroid therapy in septic shock patients [25]. However, the analysis of the impact of low-dose corticosteroids in septic shock mortality assessed in large clinical registries showed little or no effect [7] or a significant increase in mortality [26], even after adjusting for clinical severity. Similarly, a recent meta-analysis found no statistically significant difference in mortality (relative risk 1.00, 95% CI, 0.84, 1.18) [27]. Recently, Dellinger and coworkers [8] found that hydrocortisone failed to show any benefit on outcome (relative risk 1.06) if the meta-analysis included only the six high-level RCTs with low risk of bias [11,14,28-31] and excluded studies with placebo mortality >60%.

In the midst of these conflicting results, two recent observational studies were published [32,33] that brought a little light to these issues [34,35]. The first study from Katsenos *et al.* [32], showed a potential mortality benefit from early initiation of steroids (in the first 9 hours after vasopressors). However, these results are compromised by several limitations, namely the small and asymmetric sample size, the fact that the impact of steroid therapy was not adjusted for clinical severity nor organ dysfunction, and the very high mortality rate at 28 days (almost 70% in patients with late steroid initiation) [34]. The study from Funk *et al.* [33] was a large retrospective multicenter propensity-matched cohort study that showed no benefit from low-dose corticosteroids in septic shock patients either in 30-day mortality or vasopressor dependence. However, in those with higher severity, with APACHE II ≥30, there might be a benefit, whereas in the lower clinical-severity quartiles there might be potential harm. Similarly, this study has also several limitations, in particular the very long period of patient inclusion (11 years) during which a marked change in mortality was expectable [6,7,9], and the propensity score did not include variables associated with shock severity, namely the SOFA score or the number and dose of vasopressors. Finally, almost 16% of low-dose corticosteroids given to septic shock patients did not have APACHE II score recorded.

Conversely, when the two largest RCTs [11,14] of low-dose corticosteroids were analyzed, one (n = 300) suggests a marked positive impact of steroids on mortality in septic shock only in the patients who did not respond to the short corticotropin test, whereas the second (n = 499) found no beneficial effect irrespective of the response to the short corticotropin test [14].

However, these two RCTs are not totally comparable. Septic shock patients in the positive trial had a higher Simplified Acute Physiology Score II at baseline, were unresponsive to vasopressors, were all under mechanical ventilation (compared to 86% in CORTICUS and 82% in PROWESS-Shock) and there was a much higher rate of death at 28 days in the placebo group (61% compared with 32% in the CORTICUS trial). The enrolment of patients in the positive trial was allowed only within 8 hours after fulfilling inclusion criteria, as compared with a 72-hour window in the negative trial. Therefore, some authors perceive that results of the negative trial represent the randomization of patients whose clinicians decided not to treat with corticosteroids, that is, those with less severe clinical presentation [17,18]. Taken together, these findings might suggest a potential benefit of steroids for the most severe cases at the earliest stages of septic shock.

In the present analysis of PROWESS-Shock trial, we confirmed that steroid use in usual care was indeed reserved for more critically ill individuals. However, we could not confirm such benefit from this practice, even when steroids were administered early in the course of shock, with or without concomitant DrotAA. The same was true in the different subgroup analyses, namely of patients with lung infection, abdominal infection, Gram-positive infection or Gram-negative infection.

Both trials that assessed the efficacy of DrotAA [10,15] allowed the use of intravenous steroids at the discretion of the attending physician. In line with the original recommendations of the Surviving Sepsis Campaign [12], as well as the 2008 revision [13], the administration of intravenous steroids for treatment of septic shock was recommended and as a result its prescription increased from 36.0% in the original PROWESS trial to 49.5% in the PROWESS-Shock trial.

The potential synergies in the concomitant use of corticosteroids and DrotAA were evaluated in only one recent study, and this analysis was limited by the discontinuation of the DrotAA [16]. However, the authors found no significant interaction between corticosteroids and DrotAA (*P* = 0.47). Similarly, in our analysis we were unable to find any significant interaction between these two drugs among the total patient group, or in the different subgroups, namely of patients with lung infection, abdominal infection, Gram-positive or Gram-negative sepsis.

In addition, we were unable to demonstrate any significant improvement in hemodynamic stability associated with the use of corticosteroids [11,14]. Nonetheless we could not evaluate the response to the short corticotropin test, as it was not routinely performed and if performed those data were not collected in the PROWESS-Shock trial. However, in the past decade a significant amount of data have questioned the validity of the results of such a test in this setting [36,37]. First, there is a great variability of cortisol measurements observed between different methods and laboratories [36]. Also, the relationship between total and free cortisol levels had also been shown to be poor [38]. Finally, it has been shown in critically ill patients that cortisol production was 83% higher and cortisol clearance was 50% lower in comparison to matched controls. These factors account for a 3.5 times greater cortisol level in these patients [39].

In our study there are also several limitations that need to be acknowledged. The present study was not designed to stratify by the use of corticosteroids a priori and unmeasured confounders may have been missed or incompletely accounted for in our propensity adjustments. Enroled patients must have survived the initial resuscitation period to be randomized, which was on average 17 hours from the onset of vasopressor use [15]. As a result only septic shock patients that survived to that time point were analyzed, excluding very sick septic shock patients. As a result patients with early refractory shock and early deaths during this period were not included. We were unable to analyze the type, the dose of corticosteroid drug administered and the duration of steroid therapy, as these data were not collected. Only the prescription of intravenous steroid therapy for septic shock during the pretreatment period (before study drug infusion) was recorded. In addition, data on etomidate and fludrocortisone prescription was not collected in the PROWESS-Shock database. Similarly the steroid-related complications namely myopathy, nosocomial infections, and metabolic alteration were not fully available. However, if we consider endpoints such as all-cause mortality and duration of mechanical ventilation as surrogates for these complications, we did not find significant differences among the groups. Nonetheless, we acknowledge that these safety issues deserve in-depth analysis with specific and robust data collection in future studies.

The present study does have several strengths. Our analyses utilized data from a large multicenter and well-conducted RCT collected during a 25-month period and with a 90-day follow up. In addition, the inverse probability of treatment weighting using the propensity score to perform our analysis balances measured covariates between those prescribed steroids and those not prescribed steroids [23].

## Conclusions

In the present retrospective analysis of the PROWESS-Shock trial database, we were unable to find a noticeable positive impact from intravenous steroids for treatment of septic shock at baseline either in patients randomized to DrotAA or those randomized to placebo. These data could support the premise that intravenous steroids should not be systematically used in patients with septic shock; however further research in a large RCT is warranted.

## Key messages

- In the PROWESS-Shock trial, 49.5% of patients received intravenous steroids for treatment of septic shock at baseline.
- Septic shock patients treated with intravenous steroids at baseline had more organ dysfunction, higher APACHE II and SOFA scores, and needed more vasopressors.
- After adjustment for treatment selection bias, intravenous steroids for treatment of septic shock at baseline had no impact on 28-day and 90-day mortality, either in patients randomized for drotrecogin-alfa (activated) or placebo.
- The course of septic shock, assessed by the number of vasopressor-free days (propensity-weighted), was also similar in patients treated with and without intravenous steroids.
- No significant interaction between intravenous steroids and drotrecogin-alfa (activated) was found.

## Abbreviations

APACHE: acute physiology and chronic health evaluation II score
DrotAA: drotrecogin-alfa (activated)
RCT: randomized controlled trial
SOFA: sequential organ failure assessment score
